# Structural insights into lacto‐*N*‐biose I recognition by a family 32 carbohydrate‐binding module from *Bifidobacterium bifidum*

**DOI:** 10.1002/1873-3468.70217

**Published:** 2025-11-07

**Authors:** Xinzhe Zhang, Naoki Sunagawa, Toma Kashima, Kiyohiko Igarashi, Akimasa Miyanaga, Shinya Fushinobu

**Affiliations:** ^1^ Department of Biotechnology The University of Tokyo Japan; ^2^ Department of Biomaterial Sciences, Graduate School of Agricultural and Life Sciences The University of Tokyo Japan; ^3^ Collaborative Research Institute for Innovative Microbiology The University of Tokyo Japan

**Keywords:** *Bifidobacterium bifidum*, carbohydrate‐binding module family 32, crystal structure, glycoside hydrolase family 20, lacto‐*N*‐biosidase, sugar binding domain

## Abstract

*Bifidobacterium bifidum*, a predominant colonizer of the infant gut, utilizes lacto‐*N*‐biose I (LNB), a prominent component of human milk oligosaccharides (HMOs), through a dedicated metabolic pathway. Among a diverse set of extracellular glycosidases involved in HMO degradation, lacto‐*N*‐biosidase (LnbB) plays a pivotal role by releasing LNB. We investigated the structure and function of the carbohydrate‐binding module family 32 (CBM32) domain located at the C‐terminus of the glycoside hydrolase family 20 catalytic domain in LnbB. Isothermal titration calorimetry showed that CBM32 binds LNB with a dissociation constant (*K*_d_) of 98 μm. The crystal structure of the CBM32 complexed with LNB reveals the molecular basis for its specific recognition.

**Impact statement**

Bifidobacteria are beneficial gut microbes, and infant‐associated strains establish symbiosis by degrading human milk oligosaccharides. This study uncovers the molecular mechanism by which *Bifidobacterium bifidum* captures lacto‐*N*‐biose I, a key disaccharide, functioning as a cross‐feeder that promotes the growth of other bifidobacteria and supports the infant gut ecosystem.

## Graphical abstract

*Bifidobacterium bifidum* establishes symbiosis with infants by metabolizing lacto‐*N*‐biose I (LNB) from human milk oligosaccharides (HMOs). The extracellular multidomain enzyme LnbB drives this process, releasing LNB via its catalytic glycoside hydrolase family 20 (GH20) lacto‐*N*‐biosidase domain. Its carbohydrate‐binding module family 32 (CBM32) domain binds LNB (*K*_d_ = 98 μm), and the crystal structure of the CBM32–LNB complex reveals the molecular basis of recognition.

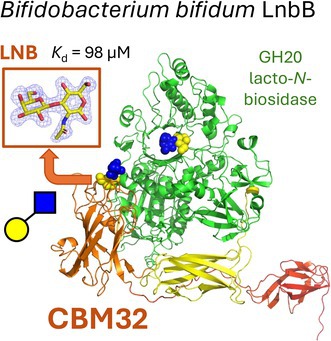

## Abbreviations

**CBM**, carbohydrate‐binding module

**GH**, glycoside hydrolase

**GNB**, galacto‐*N*‐biose

**HMO**, human milk oligosaccharide

**ITC**, isothermal titration calorimetry

**LNB**, lacto‐*N*‐biose I

**LNBase**, lacto‐*N*‐biosidase

**LnbB‐CBM32**, CBM32 of LnbB

**RMSD**, root mean square deviation

**TSA**, thermal shift assay

**βSW‐LnbB‐CBM32**, CBM32 of LnbB with β‐sandwich domain

The human gastrointestinal tract hosts complex microbial communities that begin to establish at birth. Among them, bifidobacteria confer numerous health benefits, including modulation of host‐microbiota interactions, production of bioactive metabolites, and degradation of polysaccharides inaccessible to the host [1]. In breastfed infants, *Bifidobacterium bifidum*, *B. breve*, *B. longum* subsp. *longum*, and *B. longum* subsp. *infantis* are predominant members of the gut microbiota [2, 3]. These species utilize human milk oligosaccharides (HMOs), the third most abundant solid component of human milk after lactose and lipids, as primary carbon sources [4].

HMOs are a heterogeneous mixture of over 100 structurally distinct free oligosaccharides (degree of polymerization ≥ 3), composed of galactose (Gal), glucose (Glc), *N*‐acetylglucosamine (GlcNAc), l‐fucose (Fuc), and sialic acid (Neu5Ac) [5]. They typically contain a lactose (Gal‐β1,4‐Glc) unit at the reducing end and are categorized into two major types based on the core structure at the non‐reducing end. Type I HMOs contain lacto‐*N*‐biose I (Gal‐β1,3‐GlcNAc; LNB), while type II HMOs contain *N*‐acetyllactosamine (Gal‐β1,4‐GlcNAc; LacNAc). These core structures are often modified by Fuc and Neu5Ac through α1,2/3/4‐ and α2,3/6‐linkages, respectively. Notably, type I HMOs are more abundant in human milk than type II HMOs, distinguishing it from the milk of other primates and mammals.

To utilize LNB, infant‐associated bifidobacteria have evolved the GNB/LNB pathway, a specialized metabolic system involving an ATP‐binding cassette (ABC) transporter and specific intracellular enzymes [6]. This system also imports and metabolizes galacto‐*N*‐biose (Gal‐β1,3‐GalNAc; GNB), a mucin‐derived disaccharide structurally similar to LNB. Since LNB is not present as a free disaccharide in HMOs, bifidobacteria adopt different degradation strategies by species [7]. *B. bifidum* uses an “extracellular digestion strategy,” employing various extracellular glycosidases such as fucosidases and sialidases to break down HMOs, releasing LNB and monosaccharides that are subsequently imported and metabolized [8]. This strategy enables *B. bifidum* to behave as a “cross‐feeder,” liberating host‐derived sugars that support the growth of other bifidobacterial species.

Lacto‐*N*‐biosidase (LNBase, EC3.2.1.140) is key to this process, catalyzing the cleavage of LNB from the non‐reducing end of type I HMOs. LNBase from *B. bifidum* JCM 1254 (LnbB) is a multidomain enzyme composed of 1112 amino acids and features an N‐terminal glycoside hydrolase (GH) family 20 domain and a C‐terminal carbohydrate‐binding module (CBM) family 32 domain [9]. While the crystal structure of the GH20 catalytic domain has been determined, providing insights into its substrate recognition and catalytic mechanism [10], the structural and functional properties of the CBM32 domain remain unexplored.

Here, we characterized the sugar‐binding specificity of the CBM32 domain and determined its crystal structure in both apo (ligand‐free) and LNB complex forms. We further discuss the potential role of CBM32 in facilitating HMO degradation and metabolism by *B. bifidum*, a prominent cross‐feeder in the infant gut microbiome.

## Materials and methods

### Construction of the expression plasmids

The overexpression vector was constructed for N‐terminally His_6_‐tagged CBM32 with a β‐sandwich domain (βSW‐LnbB‐CBM32, residues 670‐938) and CBM32 domain only (LnbB‐CBM32, residues 775‐938) regions of the *lnbB* gene product (GenBank accession no. EU281545.1) [9]. DNA amplification by PCR was performed using KOD One PCR Master Mix (TOYOBO Co., Ltd., Osaka, Japan). Genomic DNA was extracted from *B. bifidum* JCM 1254 cells using a Wizard Genomic DNA purification kit (Promega, Madison, WI, USA). The CBM32 regions were amplified by PCR using the genomic DNA as a template. The following primers were used for PCR: 5′‐GTGCCGCGCGGCAGCACCGGCCACATGGGCATG‐3′ (forward) and 5′‐AGCAGCCGGATCTCAAGTGGCCTTGGCCGTCG‐3′ (reverse) for βSW‐LnbB‐CBM32, and 5′‐GTGCCGCGCGGCAGCAGCCTGACCAAGGACGTGGAAG‐3′ (forward) and 5′‐AGCAGCCGGATCTCAAGTGGCCTTGGCCGTCG‐3′ (reverse) for LnbB‐CBM32. The PCR products were analyzed by agarose gel electrophoresis. The target gene fragments were extracted from the gel and used as a template for another PCR amplification with the same primer sets. The PCR products were treated with methylation‐sensitive restriction enzyme DpnI (TaKaRa Bio Inc., Shiga, Japan) and purified using a FastGene gel/PCR extraction kit (NIPPON Genetics Co., Ltd, Tokyo, Japan). Linearized vector DNA was amplified by PCR using pET28b plasmid (Novagen, Madison, WI, USA) as a template and the following primers: 5′‐TGAGATCCGGCTGCTAACAAAGCC (forward) and 5′‐GCTGCCGCGCGGCAC‐3′ (reverse). After purification using a FastGene gel/PCR extraction kit, the DNA was treated with DpnI. The CBM32 fragments were inserted into the vector DNA using the In‐Fusion HD Cloning Kit (Clontech‐Takara, Shiga, Japan) and transformed into *Escherichia coli* JM109. The expression plasmid was verified by DNA sequencing (Azenta Life Sciences, Tokyo, Japan).

### Protein production and purification

Plasmid DNA was extracted from *E. coli* JM109 using a FastGene™ plasmid mini kit (NIPPON Genetics Co., Ltd) and transformed into the overexpression host strains, *E. coli* Rosetta2 (DE3) and BL21‐CodonPlus (DE3) for βSW‐LnbB‐CBM32 and LnbB‐CBM32, respectively. The transformants were cultured in lysogeny broth medium containing 100 mg·L^−1^ kanamycin and 17 mg·L^−1^ chloramphenicol at 37 °C until the OD_600_ reached approximately 0.5. Protein expression was induced by adding isopropyl‐β‐d‐thiogalactopyranoside to a final concentration of 0.1 mm (for βSW‐LnbB‐CBM32) or 0.5 mm (for LnbB‐CBM32), and the cultures were incubated at 25 °C (for βSW‐LnbB‐CBM32) or 15 °C (for LnbB‐CBM32) for 22 h. Expression levels were analyzed by SDS/PAGE.

The cultured cells were harvested by centrifugation and suspended in 50 mm Tris–HCl (pH 7.0) and 150 mm NaCl (lysis buffer). Cell extracts were obtained by sonication, followed by centrifugation to remove cell debris. The lysate was applied to a Ni‐NTA column (Qiagen, Hilden, Germany) equilibrated with the lysis buffer. The column was washed with the lysis buffer containing 5 mm imidazole, and the His‐tagged protein was eluted with the lysis buffer containing 500 mm imidazole. Imidazole in the eluted protein sample was removed using a Vivaspin (10‐kDa or 3‐kDa cut‐off; Sartorius Stedim Biotech, Göttingen, Germany). The protein was further purified by gel filtration chromatography using a HiLoad 16/600 Superdex 75 pg column (Cytiva, Marlborough, MA, USA) equilibrated with the lysis buffer. Protein concentration was determined using both 280 nm absorbance and TaKaRa BCA Protein Assay Kit (Takara Bio Inc.) with bovine serum albumin as the standard.

### Crystallography

The crystals were obtained using the sitting‐drop vapor diffusion method by mixing equal volumes of protein and reservoir solutions. For apo crystals, a protein solution containing 20 mg·mL^−1^ LnbB‐CBM32 and a reservoir solution containing 0.1 m Tris–HCl (pH 8.0) and 3.2 m ammonium sulfate was used. For the LNB complex, a protein solution containing 20 mg·mL^−1^ LnbB‐CBM32 and 25 mm LNB and a reservoir solution containing 1.0 m LiCl, 0.1 m HEPES‐NaOH (pH 7.0), and 20% (w/v) PEG 6000 was used. The crystals were grown at 20 °C for 7–10 days and cryoprotected using the reservoir solution supplemented with glycerol at a final concentration (v/v) of 20% (for apo) or 25% (for LNB complex). Diffraction data were collected at 100 K on the beamlines at SPring‐8 (Hyogo, Japan) and the Photon Factory of the High Energy Accelerator Research Organization (KEK, Tsukuba, Japan). The X‐ray diffractions were measured using a hybrid photon counting detector installed at the beamlines (Dectris PILATUS 6M in BL45XU and Dectris EIGER X 4M in BL‐1A). The datasets were processed using xds [11] and aimless [12], or the automated data processing pipeline kamo [13]. For the apo crystal data, multiple small‐wedge (30°) data sets were clustered using blend [14], and a cluster with the lowest *R*_meas_ in the inner shell was selected. The initial phase was obtained through molecular replacement using phaser [15] and an AlphaFold2 model [16]. Manual model rebuilding and refinement were achieved using coot [17], refmac5 [18], and phenix [19]. Polder maps were prepared using phenix [20]. Anomalous difference Fourier maps were prepared using phaser [21]. Molecular graphic images were prepared using pymol (Schrödinger LLC, New York, NY, USA).

### Thermal shift assay

Thermal shift assay (TSA) was performed using the Applied Biosystems StepOne Real‐Time PCR system (Thermo Fisher Scientific Inc., Waltham, MA, USA). The purified protein samples were diluted to a final concentration of 1.0 mg·mL^−1^ (βSW‐LnbB‐CBM32) or 5.0 mg·mL^−1^ (LnbB‐CBM32) in reaction buffer containing 50 mm Tris–HCl (pH 7.0), 150 mm NaCl, with or without supplementation of LNB or GNB at different concentrations. The protein melting reaction solution (17.5 μL) was mixed with 2.5 μL Protein Thermal Shift Dye (8×), placed in a 48‐well reaction plate, and sealed. The program of thermal shift was set in continuous mode from 25 °C to 99 °C at a speed of 1% (around 0.022 °C·s^−1^), and the reaction was monitored by reporter‐type ROX. The results were analyzed to calculate melting temperature (*T*_m_) using protein thermal shift Software v1.0 (Thermo Fisher Scientific). The apparent *K*_d_ value was calculated by sigmaplot 12.0 (Grafiti LLC, Palo Alto, CA, USA) using the following equation based on the Langmuir adsorption model: *T*_m_ = Δ*T*_m_[*L*]/(*K*_d_ + [*L*]) + *B*, where [*L*] is the ligand concentration, Δ*T*_m_ is the maximum *T*_m_ change, and *B* is the offset (*T*_m_ without ligand).

### Isothermal titration calorimetry

The isothermal titration calorimetry (ITC) measurement was performed at 25 °C using Micro‐Cal VP‐ITC (Malvern Instruments Ltd., Malvern, Worcestershire, UK). Purified LnbB‐CBM32 protein dissolved in 50 mm Tris–HCl buffer (pH 7.0) was dialyzed three times using Slide‐A‐Lyzer G3 Dialysis Cassettes (3K MWCO; Thermo Fisher Scientific). Ligand solutions were prepared by dissolution with the same buffer obtained from the external fluid after dialysis. The protein solution (0.49 mm) was stirred at 307 r.p.m. in a 1.43 mL cell. The first titration proceeded with 2 μL for 4 s. After a 600 s interval, the second to 52nd titrations of 5 μL proceeded for 10 s at intervals of 600 s. Ligand concentrations were 10 mm for LNB, GNB, and Gal. Calorimetric data were analyzed using origin 7.0 software (LightStone Corp., New York, NY, USA). Thermodynamic parameters, such as association constants (*K*_a_), binding enthalpy (Δ*H*), and the number of binding sites (*n*), were determined by fitting data into a one‐site binding model. Changes in Gibbs binding free energy (Δ*G* ^0^), dissociation constants (*K*_d_), and binding entropy changes (Δ*S* ^0^) were calculated from the equations: Δ*G* ^0^ = –*RT*ln*K*_a_ = *RT*ln*K*_d_ and Δ*G* ^0^ = Δ*H* − *T*Δ*S* ^0^, where *R* and *T* are the gas constant and absolute temperature (298.15 K), respectively. We assumed that Δ*H* values determined from ITC equal the standard enthalpy change (Δ*H* ^0^).

## Results

### Protein constructs and stability analysis

**Fig. 1:**
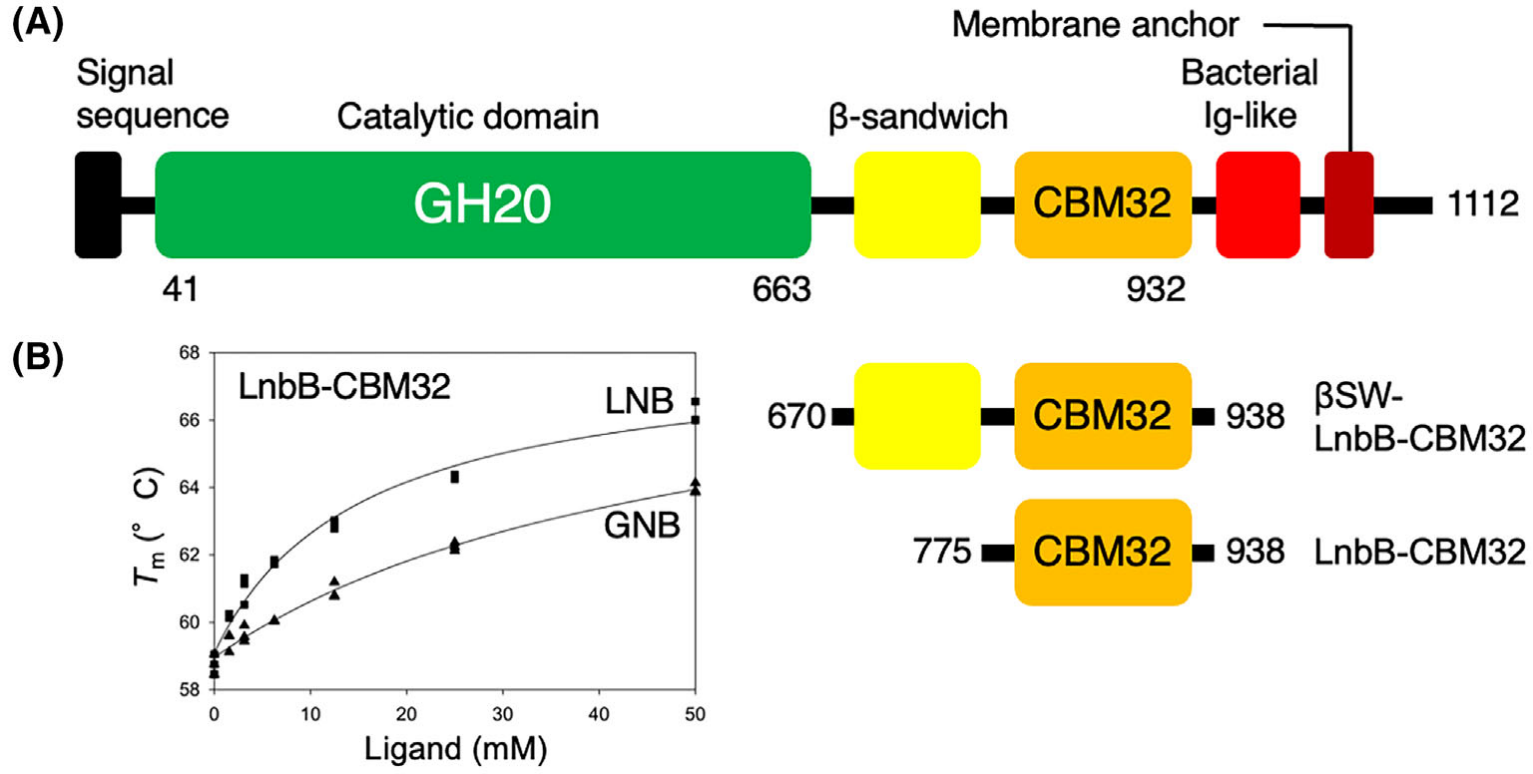
Domain architecture of *Bifidobacterium bifidum* lacto‐*N*‐biosidase (LnbB) and thermal stability analysis of its carbohydrate‐binding module (CBM) family 32 domain. (A) Schematic representation of the full‐length domain architecture of LnbB. Two truncated constructs used for this study are shown:βSW‐LnbB‐CBM32 containing the β‐sandwich and CBM32 domains, and LnbB‐CBM32 containing only the CBM32 domain. (B) *T*_m_ shift of LnbB‐CBM32 in the presence of lacto‐*N*‐biose I (LNB) (squares) or galacto‐*N*‐biose (GNB, triangles), as measured by thermal shift assay (TSA). The melting curves are shown in Fig. S3. The data points (*n*) were 20 for both LNB and GNB.

LnbB comprises a signal peptide, a catalytic GH20 domain, a β‐sandwich domain, a CBM32 domain, a bacterial immunoglobulin (Ig)‐like domain, and a C‐terminal transmembrane region (Fig. 1A and Fig. S1A). Structural prediction using AlphaFold3 [22] suggested that the CBM32 and preceding β‐sandwich domains are spatially separated from the GH20 domain, and the predicted aligned error plot indicated that the β‐sandwich and CBM32 domains have weak interactions (Fig. S1B). To investigate the function of CBM32, two recombinant constructs were generated: one including the β‐sandwich domain (βSW‐LnbB‐CBM32) and one comprising only the CBM32 domain (LnbB‐CBM32) (Fig. 1A).

Both proteins were successfully purified and appeared as single bands on SDS/PAGE, corresponding to their theoretical molecular masses of 30 011 and 19 423 Da, respectively (Fig. S2A). LnbB‐CBM32 was confirmed to be monomeric in solution as determined by size exclusion chromatography (Fig. S2B). TSA revealed that the melting temperature (*T*_m_) of LnbB‐CBM32 increased by ~ 7 °C upon the addition of 50 mm LNB, and by ~ 5 °C with 50 mm GNB (Fig. S3). Based on concentration‐dependent *T*_m_ shifts, the apparent dissociation constants (*K*_d_) and maximum *T*_m_ change (Δ*T*_m_) for LNB and GNB were estimated as 15.2 ± 2.5 mm and 8.92 ± 0.47 °C, and 49.9 ± 10.9 mm and 9.95 ± 1.16 °C, respectively (Fig. 1B). In contrast, βSW‐LnbB‐CBM32 exhibited no significant *T*_m_ changes upon ligand binding (Fig. S4), suggesting altered or reduced ligand‐binding properties compared to LnbB‐CBM32. Based on these results, we selected LnbB‐CBM32 for further analysis by ITC and crystallography.

### Determination of carbohydrate‐binding affinity using ITC

**Fig. 2:**
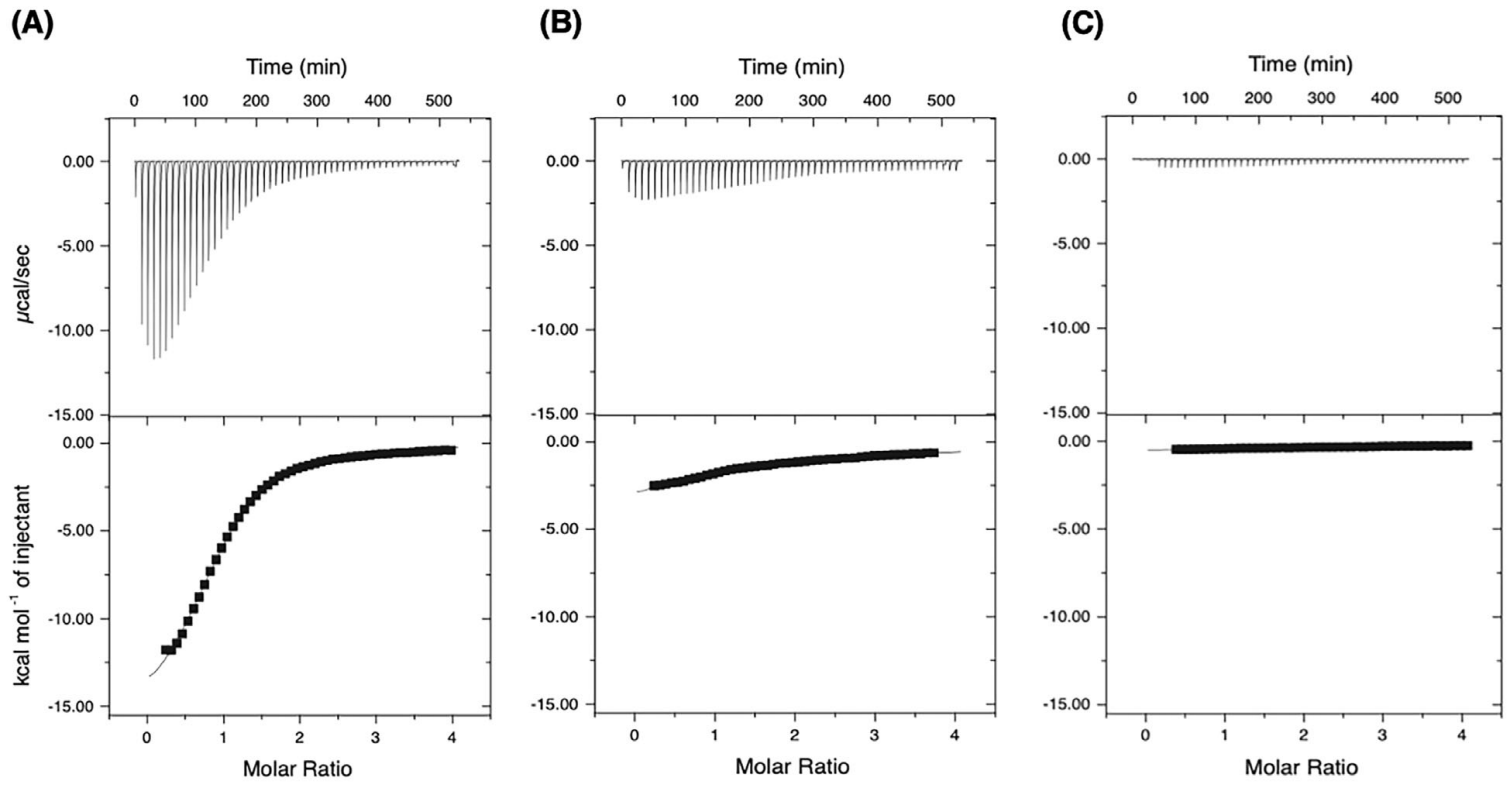
ITC analysis of LnbB‐CBM32 binding to carbohydrates. Thermograms and binding isotherms obtained at 25 °C for LnbB‐CBM32 titrated with (A) LNB, (B) GNB, and (C) galactose (Gal) are shown. The top panels show the heat changes per injection, and the bottom panels show the integrated heat plotted against the molar ratio of ligand to protein. Fitting to a one‐site binding model was performed, and the resulting thermodynamic parameters are summarized in Table 1.

The *K*_d_ values estimated by TSA can be inaccurate for measuring the ligand affinity of native proteins, as the method involves irreversible protein denaturation [23]. To obtain more reliable affinity and thermodynamic parameters, we performed ITC on LnbB‐CBM32 with carbohydrate ligands. The binding isotherms for LNB and GNB showed typical sigmoidal curves, with LNB releasing more heat than GNB (Fig. 2). In contrast, titration with Gal produced a flat line, indicating no specific binding. One‐site binding model curve fitting yielded *K*_d_ values of 98 μm for LNB and 1.4 mm for GNB (Table 1). Both interactions were enthalpy‐driven, exhibiting large negative Δ*H* values, suggesting that hydrogen bonding predominantly mediates ligand recognition.

**Table 1:** Affinity and thermodynamic parameters of ligand binding to LnbB‐CBM32 at 25 °C measured by ITC.

Ligand	LNB	GNB
*K*_a_ (×10^3^ m ^−1^)	10.2 ± 0.4	0.711 ± 0.070
*K*_d_ (μm)	98.0	1410
*n*	0.921 ± 0.010	0.749 ± 0.187
Δ*G* ^0^ (kcal·mol^−1^)	−5.47	−3.89
Δ*H* (kcal·mol^−1^)	−16.2 ± 0.2	−14.0 ± 3.9
−*T*Δ*S* (kcal·mol^−1^)	10.9	10.1
*c*	4.60	0.260

### Crystal structure

**Fig. 3:**
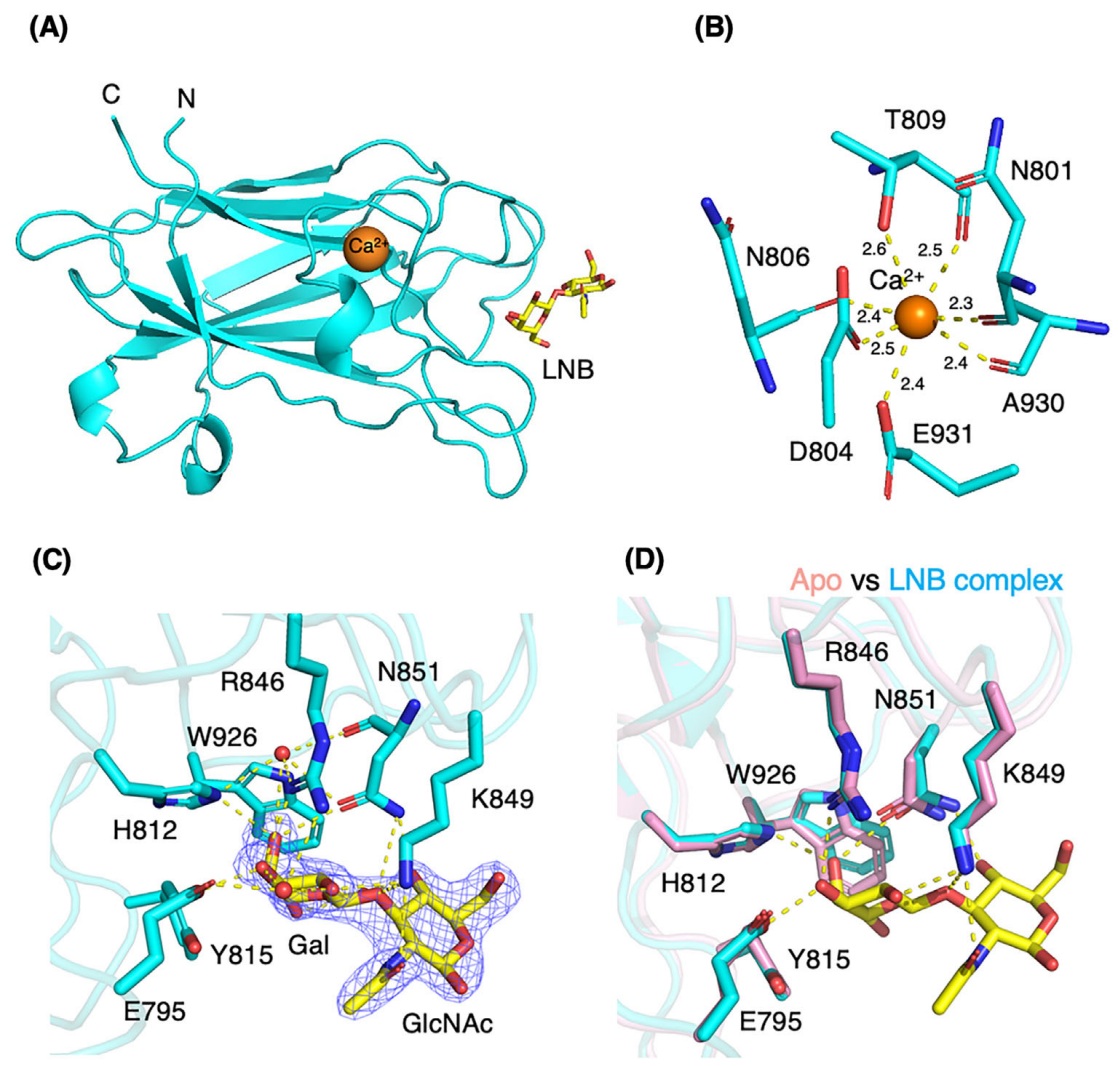
Crystal structure of LnbB‐CBM32 in complex with LNB. (A) The overall structure of LnbB‐CBM32 (cyan) complexed with LNB (yellow sticks) and a calcium ion (orange sphere). (B) Close‐up view of the Ca^2+^‐binding site. The coordination distances in Å are shown. (C) LNB‐binding site with a Polder map (blue mesh, 3σ). (D) Superposition of the apo (pink) and LNB complex (cyan) structures, highlighting conformational changes at the ligand‐binding site. Hydrogen bonds and metal coordination are shown as yellow dashed lines.

The crystal structures of LnbB‐CBM32 were determined in both apo and LNB‐bound forms at 2.00 Å resolution (Table S1). The apo crystal contained two LnbB‐CBM32 molecules in the asymmetric unit, while the LNB complex crystal contained four molecules. All six protein molecules in the asymmetric units of two crystals were virtually identical in overall structure, with the only difference around the sugar ligand binding site. The root mean square deviations (RMSD) for Cα atoms were less than 0.290 Å between all pairs of molecules within both the apo and LNB complex crystals. The overall fold adopts a typical β‐sandwich architecture, comprising an 8‐stranded antiparallel β‐sheet flanked by three short helices (Fig. 3A).

A calcium ion is hepta‐coordinated within the protein, involving side chains of D804, T809, and E931, as well as the main chain carbonyl groups of N801, N806, T809, and A930 (Fig. 3B). Notably, the Ca^2+^ ion interacts simultaneously with both the main chain and side chain of T809. Analysis using the CheckMyMetal server confirmed that the coordination distances and geometry are consistent with canonical Ca^2+^ binding, and not compatible with Mn^2+^, Mg^2+^, or other metal‐binding profiles [24]. Weak anomalous difference map signals were observed at most sites in the asymmetric units of crystals (Fig. S5), consistent with the data collection wavelengths (1.000 and 1.012 Å), which are shorter than the absorption edge of calcium (3.0704 Å). Similar calcium‐binding sites are conserved across other CBM32 family proteins, as discussed below.

The crystal structure of LnbB‐CBM32 complexed with LNB revealed the detailed interactions responsible for ligand binding. LNB was bound in a cleft‐like shallow groove (Fig. 3A). Among the four chains, LNB in chain A exhibited the clearest electron density with β‐anomer at the reducing end GlcNAc (Fig. S6). Therefore, we focused on the site in chain A. Both the Gal and GlcNAc moieties of LNB adopt the stable ^4^ *C*_1_ chair conformation (Fig. 3C). The Gal moiety is stabilized by CH‐π interactions with the aromatic side chains of Y815 and W926 and forms multiple hydrogen bonds with E795, H812, R846, and K849, as well as two water molecules. The GlcNAc moiety forms hydrogen bonds with K849 and N851, and its *N*‐acetyl group adopts an extended conformation. The equatorial O4 hydroxy group of GlcNAc forms a hydrogen bond with N851, an interaction that would be lost if GlcNAc were replaced with GalNAc. This structural difference likely accounts for the 2.2 kcal·mol^−1^ enthalpy difference observed in ITC between LNB and GNB binding. The axial O4‐hydroxy group of GalNAc in GNB may also cause steric hindrance with the side chain of K849.

Comparison of the apo and LNB‐bound structures revealed that the positions of most ligand‐binding residues remained largely unchanged, except for N851 and W926 (Fig. 3D). The side chain of W926, which forms a hydrophobic pocket with Y815, slightly adjusts its orientation to optimize the interaction with the Gal moiety. The water molecules involved in the hydrogen bonding network, reorganized at the binding site, aid in LNB recognition and probably contribute to the negative entropy changes as measured by ITC experiments (Table 1).

## Discussion

### Structural comparison with other CBM32 proteins

A Dali structural similarity search revealed that LnbB‐CBM32 shares a common fold with CBM32 domains from GH84 *N*‐acetyl‐β‐hexosaminidase, AA5_2 galactose oxidase, GH33 sialidase, and GH29 α‐fucosidase, despite low sequence identities (< 30%) (Table S2). Among these, we focused on comparison with the CBM32 domain from *Clostridium perfringens N*‐acetyl‐β‐hexosaminidase GH84C (*Cp*CBM32; PDB ID: 2J1A) [25], which showed the highest structural similarity. Other CBM32 structures with certain structural similarity (*Z* score ≥ 16.5) do not bind any ligands.

**Fig. 4:**
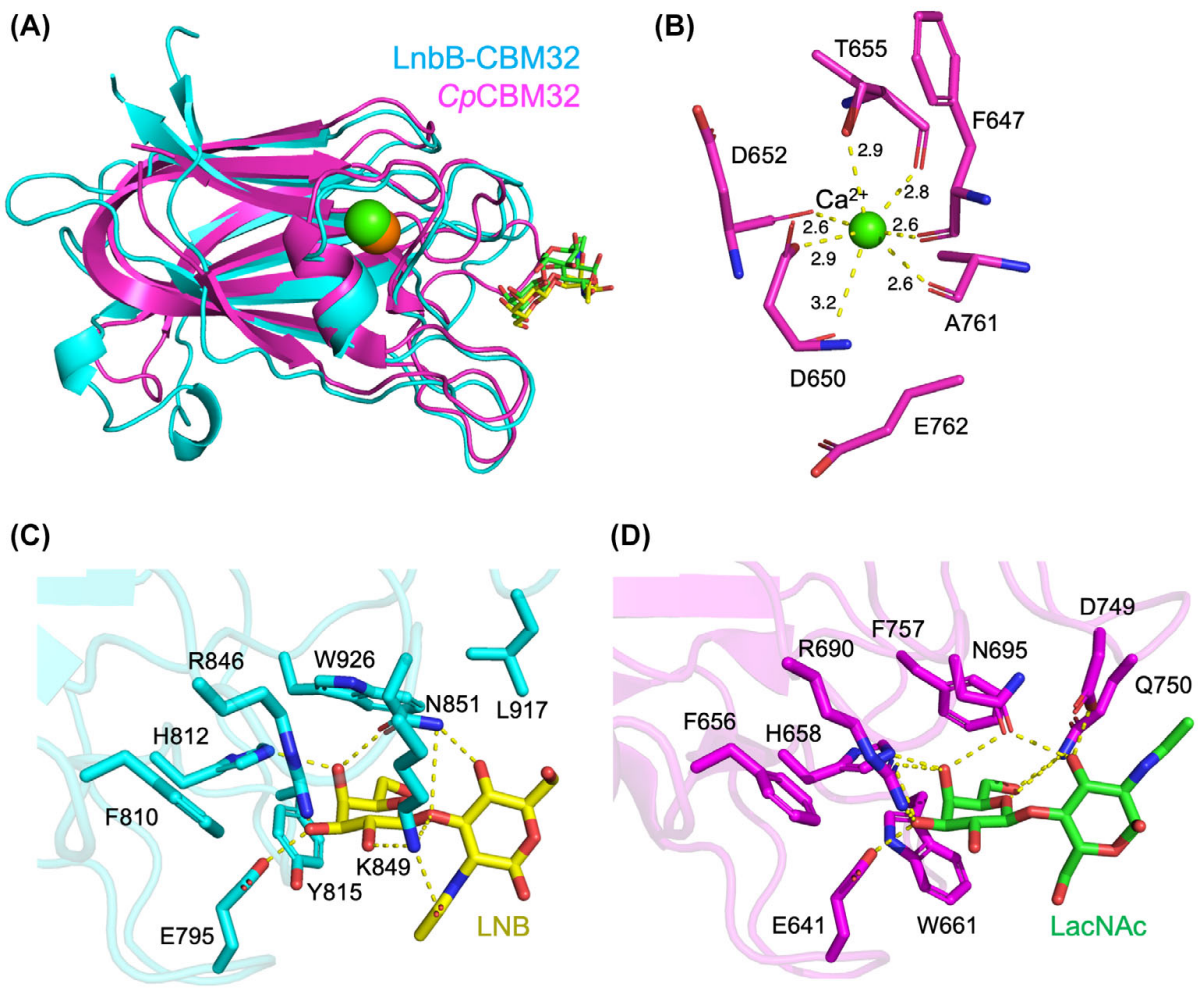
Structural comparison of LnbB‐CBM32 (cyan) complexed with LNB (yellow sticks) and CBM32 domain from *Clostridium perfringens N*‐acetyl‐β‐hexosaminidase GH84C (*Cp*CBM32, magenta, PDB ID: 2J1E) complexed with *N*‐acetyllactosamine (LacNAc, green sticks). (A) Superposition of the overall structures. Calcium ions in LnbB‐CBM32 and *Cp*CBM32 are shown as orange and green spheres, respectively. (B) Close‐up view of the calcium‐binding site in *Cp*CBM32. The coordination distances in Å are shown. (C) Ligand‐binding site of LnbB‐CBM32. (D) Ligand‐binding site of *Cp*CBM32. Hydrogen bonds and metal coordination are shown as yellow dashed lines.

Superimposition of LnbB‐CBM32 with *Cp*CBM32 complexed with LacNAc (PDB ID: 2J1E) revealed a high degree of structural similarity, including conserved positioning of the carbohydrate‐binding and calcium‐binding sites (Fig. 4A). The hepta‐coordinated calcium ion in *Cp*CBM32 is stabilized like that in LnbB‐CBM32 (Fig. 4B). However, in *Cp*CBM32, a glutamate residue (E762) is displaced from the coordination sphere, and instead, both the main chain and side chain atoms of an aspartate residue (D650) coordinate the calcium ion.

The ligand‐binding sites of LnbB‐CBM32 and *Cp*CBM32 were compared in detail (Fig. 4C,D). In the Gal‐binding pocket, E641, F656, H658, R690, and N695 of *Cp*CBM32 are conserved and engaged in similar hydrogen bonding and hydrophobic interactions as their counterparts in LnbB‐CBM32. However, the aromatic stacking residues that sandwich the Gal moiety differ: *Cp*CBM32 utilizes W661 and F757, while LnbB‐CBM32 features a tyrosine and a tryptophan at these positions. In contrast, significant divergence was observed in GlcNAc recognition, reflecting the different glycosidic linkages in the two disaccharides: β1,3 in LNB and β1,4 in LacNAc. The GlcNAc moiety in LacNAc adopts an inverted orientation compared to that of LNB. In *Cp*CBM32, the O3 hydroxy group of GlcNAc forms hydrogen bonds with the side chains of D749 and Q750. Notably, a conserved asparagine residue (N851 in LnbB‐CBM32 and N695 in *Cp*CBM32) bridges the Gal and GlcNAc units via hydrogen bonding in both proteins, although their side chain orientations differ. In LnbB‐CBM32, both the side chain nitrogen and oxygen atoms of N851 participate in ligand coordination, whereas in *Cp*CBM32, the amide oxygen of N695 is involved in bridging the two sugar moieties. Although further experimental validation is required, these structural similarities suggest that LnbB‐CBM32 may also accommodate LacNAc binding, similarly to *Cp*CBM32.

*Cp*CBM32 was also reported to bind the type II blood group H‐trisaccharide (Fuc‐α1,2‐Gal‐β1,4‐GlcNAc), albeit with lower affinity than LacNAc [25]. In its crystal structure, the fucose moiety engages in only two water‐mediated hydrogen bonds with the protein. If LacNAc were to bind LnbB‐CBM32 similarly, α1,2‐linked fucose on the O2 position of Gal might also be accommodated. However, the side chain of K849 in LnbB‐CBM32 could present steric hindrance (Fig. 4C). This lysine residue, which forms a hydrogen bond with the β1,3‐linked GlcNAc in LNB, is absent in *Cp*CBM32. These structural distinctions suggest that LnbB‐CBM32 exhibits specificity for LNB, while *Cp*CBM32 favors LacNAc. Interestingly, the differences in binding specificity align with the functional roles of their associated catalytic domains and biological contexts. The *N*‐acetyl‐β‐hexosaminidase GH84C from the pathogen *C. perfringens* is thought to scavenge carbohydrates in the human gut, producing gastric mucins, and *Cp*CBM32 was suggested to catch the terminal LacNAc motif common to the *O*‐linked glycans of mucin [25]. In contrast, LnbB from the symbiotic bacterium *B. bifidum* plays a pivotal role in human milk oligosaccharide degradation [26], and its GH20 catalytic domain exhibits strict specificity toward the unmodified β‐linked LNB motif [9, 10]. CBM32 domains, often classified within the F5/8 type C domain family, have historically been described as Gal‐binding modules based on studies of galactose oxidase [27] and GH33 sialidases [28, 29]. However, as demonstrated for LnbB‐CBM32, Gal alone does not provide sufficient interaction to support specific binding. Instead, additional contacts in the second subsite are critical for recognizing disaccharide ligands (e.g., LNB vs GNB), thereby conferring specificity beyond simple Gal recognition.

### Conserved calcium‐binding site in the CBM32 family

Calcium ion coordination is a conserved feature among CBM32 domains. For instance, CBM32 modules from *Fusarium* sp. galactose oxidase [30] and *Akkermansia muciniphila* GH29 α‐fucosidase [31] exhibit calcium‐binding sites highly similar to that of LnbB‐CBM32, typically adopting either a hepta‐ or hexa‐coordinated geometry. Among the calcium‐binding CBM32 domains, a conserved threonine residue (occasionally replaced by serine) is commonly observed to coordinate the calcium ion via both its main chain carbonyl and side chain hydroxy groups [25, 30, 31, 32, 33].

In LnbB‐CBM32, calcium is present in both apo and ligand‐bound crystal structures, indicating that its primary role is structural stabilization rather than direct involvement in ligand recognition. This observation is consistent with other CBM32 domains where calcium binding contributes to protein folding and stability.

An exception to this pattern is found in the CBM32 domain of the GH33 sialidase from *Micromonospora viridifaciens*, where a sodium ion (Na^+^) is reported to occupy the corresponding metal‐binding site [28]. However, analysis using the CheckMyMetal server suggests that the observed 7‐coordinate pentagonal bipyramidal geometry is atypical for sodium, and calcium was the more plausible metal–ligand in this position.

### A proposed role of LnbB‐CBM32 in LNB product capture and transport

CBMs are generally thought to function by binding to (often insoluble) polymeric substrates to enhance the accessibility of catalytic domains, and CBM32s are classified as “lectin‐like” type C CBMs that bind to small sugar units [34]. In *B. bifidum*, at least 13 CBM32s are associated with extracellular glycosidases and are thought to support binding to dietary polysaccharides and mucin *O*‐glycans, thereby enhancing enzymatic access to substrates [35]. However, LnbB presents an exceptional case. While the core 1 structure of mucin *O*‐glycan is identical to GNB, the GH20 domain of LnbB shows only ~ 30% of the activity toward *p*‐nitrophenyl‐GNB compared with *p*‐nitrophenyl‐LNB [9], and LnbB‐CBM32 shows significantly weaker binding to GNB than to LNB (this study). These results suggest that LnbB plays only a minor role in mucin glycan degradation.

Instead, LnbB plays a major role in the degradation of human milk oligosaccharides (HMOs), particularly type I HMOs in the infant's gut [7]. Its typical substrate, lacto‐*N*‐tetraose (Gal‐β1,3‐GlcNAc‐β1,3‐Gal‐β1,4‐Glc), is a major HMO component [5]. However, unlike typical CBM substrates, HMOs are free, soluble oligosaccharides in milk rather than insoluble or cell‐associated glycans. This raises questions about how LnbB‐CBM32 improves substrate accessibility, unlike CBMs associated with plant polysaccharides.

**Fig. 5:**
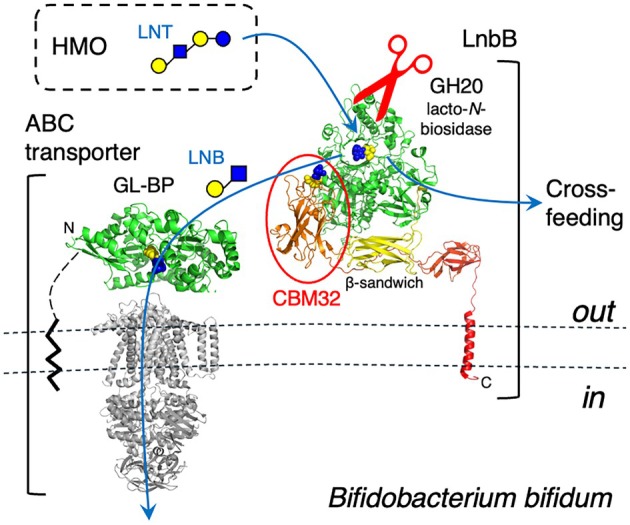
A proposed role of LnbB‐CBM32 domain in the product capture and transport. Ribbon models of LnbB and GNB/LNB‐specific ATP‐binding cassette (ABC)‐transporter (substrate‐binding protein in green, transmembrane and ATPase domains in gray) are shown. LnbB is anchored to the *Bifidobacterium bifidum* cell by a C‐terminal transmembrane helix, and the substrate‐binding protein (GNB/LNB‐binding protein, GL‐BP) is membrane‐anchored by a lipid group. The N‐terminus of LnbB and the C‐terminus of GL‐BP are at the back of the molecule. The LNB molecules (galactose in yellow and *N*‐acetylglucosamine in blue) bound to the catalytic and CBM32 domains in LnbB and to the capture site of GL‐BP are shown as spheres. A part of the sugars cleaved and released by the extracellular glycosidases of *B. bifidum* is cross‐fed to other bifidobacteria.

We hypothesize that the CBM32 domain may function not in substrate binding but in product capture. LnbB has a transmembrane anchor at its C‐terminus, placing the CBM32 domain closer to the membrane than the GH20 catalytic domain (Fig. 5). Infant gut‐associated bifidobacteria, including *B. bifidum*, possess an ABC‐type transporter specific for LNB and GNB [36]. The LnbB‐CBM32 domain may help trap the liberated LNB product near the cell surface, preventing its diffusion and efficiently channeling it to the substrate‐binding protein of the ABC transporter. Such a role would explain both the observed LNB‐binding specificity and the structural positioning of CBM32 in the domain architecture of LnbB. This mechanism may increase the efficiency of LNB uptake, supporting bifidobacterial colonization in the infant gut and contributing to host‐microbe symbiosis.

## Author contributions

SF conceived and supervised the study; NS, TK, KI, and AM designed the experiments; XZ and NS performed experiments; XZ performed protein crystallography; XZ, NS, and SF analyzed data; XZ and SF wrote the manuscript; all authors reviewed the manuscript.

## Supporting information


**Fig. S1.** Domain architecture of full‐length LnbB and a predicted structure by AlphaFold3.
**Fig. S2**. Purification of LnbB‐CBM32.
**Fig. S3**. TSA of LnbB‐CBM32 in the presence of various concentrations of LNB or GNB.
**Fig. S4**. TSA of βSW‐LnbB‐CBM32 in the presence of various concentrations of LNB or GNB.
**Fig. S5**. Anomalous difference Fourier maps.
**Fig. S6**. Electron density map for LNB.
**Table S1**. Crystallographic data statistics of LnbB‐CBM32.
**Table S2**. Result of DALI structural similarity search.

## Data Availability

Atomic coordinates and structure factors of the crystal structures have been deposited in the Protein Data Bank under accession numbers 9WFH and 9VAK. Source data are provided with this paper.

## References

[1] Bottacini F , Ventura M , van Sinderen D and Motherway MOC (2014) Diversity, ecology and intestinal function of bifidobacteria. Microb Cell Fact 13, 1–15. 25186128 10.1186/1475-2859-13-S1-S4 PMC4155821

[2] Turroni F , Peano C , Pass DA , Foroni E , Severgnini M , Claesson MJ , Kerr C , Hourihane J , Murray D , Fuligni F *et al*. (2012) Diversity of bifidobacteria within the infant gut microbiota. PLoS One 7, e36957. 22606315 10.1371/journal.pone.0036957 PMC3350489

[3] Tannock GW , Lawley B , Munro K , Pathmanathan SG , Zhou SJ , Makrides M , Gibson RA , Sullivan T , Prosser CG , Lowry D *et al*. (2013) Comparison of the compositions of the stool microbiotas of infants fed goat milk formula, cow milk‐based formula, or breast milk. Appl Environ Microbiol 79, 3040–3048. 23455335 10.1128/AEM.03910-12 PMC3623157

[4] Lawson MAE , O'Neill IJ , Kujawska M , Gowrinadh Javvadi S , Wijeyesekera A , Flegg Z , Chalklen L and Hall LJ (2020) Breast milk‐derived human milk oligosaccharides promote *Bifidobacterium* interactions within a single ecosystem. ISME J 14, 635–648. 31740752 10.1038/s41396-019-0553-2 PMC6976680

[5] Urashima T , Ajisaka K , Ujihara T and Nakazaki E (2025) Recent advances in the science of human milk oligosaccharides. BBA Adv 7, 100136. 39991261 10.1016/j.bbadva.2024.100136 PMC11847054

[6] Kitaoka M (2012) Bifidobacterial enzymes involved in the metabolism of human milk oligosaccharides. Adv Nutr 3, 422S–429S. 22585921 10.3945/an.111.001420 PMC3649479

[7] Katayama T (2016) Host‐derived glycans serve as selected nutrients for the gut microbe: human milk oligosaccharides and bifidobacteria. Biosci Biotechnol Biochem 80, 621–632. 26838671 10.1080/09168451.2015.1132153

[8] Sakanaka M , Gotoh A , Yoshida K , Odamaki T , Koguchi H , Xiao JZ , Kitaoka M and Katayama T (2020) Varied pathways of infant gut‐associated *Bifidobacterium* to assimilate human milk oligosaccharides: prevalence of the gene set and its correlation with bifidobacteria‐rich microbiota formation. Nutrients 12, 71. 10.3390/nu12010071 PMC7019425 31888048

[9] Wada J , Ando T , Kiyohara M , Ashida H , Kitaoka M , Yamaguchi M , Kumagai H , Katayama T and Yamamoto K (2008) *Bifidobacterium bifidum* lacto‐N‐biosidase, a critical enzyme for the degradation of human milk oligosaccharides with a type 1 structure. Appl Environ Microbiol 74, 3996–4004. 18469123 10.1128/AEM.00149-08 PMC2446520

[10] Ito T , Katayama T , Hattie M , Sakurama H , Wada J , Suzuki R , Ashida H , Wakagi T , Yamamoto K , Stubbs KA *et al*. (2013) Crystal structures of a glycoside hydrolase family 20 lacto‐*N*‐biosidase from *Bifidobacterium bifidum* . J Biol Chem 288, 11795–11806. 23479733 10.1074/jbc.M112.420109 PMC3636868

[11] Kabsch W (2010) XDS. Acta Crystallogr D Biol Crystallogr 66, 125–132. 20124692 10.1107/S0907444909047337 PMC2815665

[12] Evans PR and Murshudov GN (2013) How good are my data and what is the resolution? Acta Crystallogr D Biol Crystallogr 69, 1204–1214. 23793146 10.1107/S0907444913000061 PMC3689523

[13] Yamashita K , Hirata K and Yamamoto M (2018) KAMO: towards automated data processing for microcrystals. Acta Crystallogr D Struct Biol 74, 441–449. 29717715 10.1107/S2059798318004576 PMC5930351

[14] Foadi J , Aller P , Alguel Y , Cameron A , Axford D , Owen RL , Armour W , Waterman DG , Iwata S and Evans G (2013) Clustering procedures for the optimal selection of data sets from multiple crystals in macromolecular crystallography. Acta Crystallogr D Biol Crystallogr 69, 1617–1632. 23897484 10.1107/S0907444913012274 PMC3727331

[15] McCoy AJ , Grosse‐Kunstleve RW , Adams PD , Winn MD , Storoni LC and Read RJ (2007) Phaser crystallographic software. J Appl Cryst 40, 658–674. 19461840 10.1107/S0021889807021206 PMC2483472

[16] Jumper J , Evans R , Pritzel A , Green T , Figurnov M , Ronneberger O , Tunyasuvunakool K , Bates R , Žídek A , Potapenko A *et al*. (2021) Highly accurate protein structure prediction with AlphaFold. Nature 596, 583–589. 34265844 10.1038/s41586-021-03819-2 PMC8371605

[17] Emsley P , Lohkamp B , Scott WG and Cowtan K (2010) Features and development of Coot. Acta Crystallogr D Biol Crystallogr 66, 486–501. 20383002 10.1107/S0907444910007493 PMC2852313

[18] Murshudov GN , Skubák P , Lebedev AA , Pannu NS , Steiner RA , Nicholls RA , Winn MD , Long F and Vagin AA (2011) REFMAC5 for the refinement of macromolecular crystal structures. Acta Crystallogr D Biol Crystallogr 67, 355–367. 21460454 10.1107/S0907444911001314 PMC3069751

[19] Afonine PV , Poon BK , Read RJ , Sobolev OV , Terwilliger TC , Urzhumtsev A and Adams PD (2018) Real‐space refinement in PHENIX for cryo‐EM and crystallography. Acta Crystallogr D Struct Biol 74, 531–544. 29872004 10.1107/S2059798318006551 PMC6096492

[20] Liebschner D , Afonine PV , Moriarty NW , Poon BK , Sobolev OV , Terwilliger TC and Adams PD (2017) Polder maps: improving OMIT maps by excluding bulk solvent. Acta Crystallogr D Struct Biol 73, 148–157. 28177311 10.1107/S2059798316018210 PMC5297918

[21] Read RJ and McCoy AJ (2011) Using SAD data in Phaser. Acta Crystallogr D Biol Crystallogr 67, 338–344. 21460452 10.1107/S0907444910051371 PMC3069749

[22] Abramson J , Adler J , Dunger J , Evans R , Green T , Pritzel A , Ronneberger O , Willmore L , Ballard AJ , Bambrick J *et al*. (2024) Accurate structure prediction of biomolecular interactions with AlphaFold 3. Nature 630, 493–500. 38718835 10.1038/s41586-024-07487-w PMC11168924

[23] Miyake M , Terada T , Shimokawa M , Sugimoto N , Arakawa T , Shimizu K , Igarashi K , Fujita K and Fushinobu S (2020) Structural analysis of β‐L‐arabinobiose‐binding protein in the metabolic pathway of hydroxyproline‐rich glycoproteins in *Bifidobacterium longum* . FEBS J 287, 5114–5129. 32246585 10.1111/febs.15315

[24] Zheng H , Cooper DR , Porebski PJ , Shabalin IG , Handing KB and Minor W (2017) CheckMyMetal: a macromolecular metal‐binding validation tool. Acta Crystallogr D Struct Biol 73, 223–233. 28291757 10.1107/S2059798317001061 PMC5349434

[25] Ficko‐Blean E and Boraston AB (2006) The interaction of a carbohydrate‐binding module from a *Clostridium perfringens N*‐acetyl‐β‐hexosaminidase with its carbohydrate receptor. J Biol Chem 281, 37748–37757. 16990278 10.1074/jbc.M606126200

[26] Fushinobu S (2010) Unique sugar metabolic pathways of Bifidobacteria. Biosci Biotechnol Biochem 74, 2374–2384. 21150123 10.1271/bbb.100494

[27] Ito N , Phillips SEV , Stevens C , Ogel ZB , McPherson MJ , Keen JN , Yadav KDS and Knowles PF (1991) Novel thioether bond revealed by a 1.7 Å crystal structure of galactose oxidase. Nature 350, 87–90. 2002850 10.1038/350087a0

[28] Gaskell A , Crennell S and Taylor G (1995) The three domains of a bacterial sialidase: a β‐propeller, an immunoglobulin module and a galactose‐binding jelly‐roll. Structure 3, 1197–1205. 8591030 10.1016/s0969-2126(01)00255-6

[29] Newstead SL , Watson JN , Bennet AJ and Taylor G (2005) Galactose recognition by the carbohydrate‐binding module of a bacterial sialidase. Acta Crystallogr D Biol Crystallogr 61, 1483–1491. 16239725 10.1107/S0907444905026132

[30] Firbank SJ , Rogers MS , Wilmot CM , Dooley DM , Halcrow MA , Knowles PF , McPherson MJ and Phillips SEV (2001) Crystal structure of the precursor of galactose oxidase: an unusual self‐processing enzyme. Proc Natl Acad Sci USA 98, 12932–12937. 11698678 10.1073/pnas.231463798 PMC60802

[31] Shuoker B , Pichler MJ , Jin C , Sakanaka H , Wu H , Gascueña AM , Liu J , Nielsen TS , Holgersson J , Nordberg Karlsson E *et al*. (2023) Sialidases and fucosidases of *Akkermansia muciniphila* are crucial for growth on mucin and nutrient sharing with mucus‐associated gut bacteria. Nat Commun 14, 1–16. 37005422 10.1038/s41467-023-37533-6 PMC10067855

[32] Ndeh D , Rogowski A , Cartmell A , Luis AS , Baslé A , Gray J , Venditto I , Briggs J , Zhang X , Labourel A *et al*. (2017) Complex pectin metabolism by gut bacteria reveals novel catalytic functions. Nature 544, 65–70. 28329766 10.1038/nature21725 PMC5388186

[33] Cartmell A , Muñoz‐Muñoz J , Briggs JA , Ndeh DA , Lowe EC , Baslé A , Terrapon N , Stott K , Heunis T , Gray J *et al*. (2018) A surface endogalactanase in *Bacteroides thetaiotaomicron* confers keystone status for arabinogalactan degradation. Nat Microbiol 3, 1314–1326. 30349080 10.1038/s41564-018-0258-8 PMC6217937

[34] Armenta S , Moreno‐Mendieta S , Sánchez‐Cuapio Z , Sánchez S and Rodríguez‐Sanoja R (2017) Advances in molecular engineering of carbohydrate‐binding modules. Proteins 85, 1602–1617. 28547780 10.1002/prot.25327

[35] Katoh T , Ojima MN , Sakanaka M , Ashida H , Gotoh A and Katayama T (2020) Enzymatic adaptation of *Bifidobacterium bifidum* to host glycans, viewed from glycoside hydrolases and carbohydrate‐binding modules. Microorganisms 8, 481. 32231096 10.3390/microorganisms8040481 PMC7232152

[36] Suzuki R , Wada J , Katayama T , Fushinobu S , Wakagi T , Shoun H , Sugimoto H , Tanaka A , Kumagai H , Ashida H *et al*. (2008) Structural and thermodynamic analyses of solute‐binding protein from *Bifidobacterium longum* specific for core 1 disaccharide and lacto‐*N*‐biose I. J Biol Chem 283, 13165–13173. 18332142 10.1074/jbc.M709777200

