# Supplementary material for: Structural insights into lacto‐N‐biose I recognition by a family 32 carbohydrate‐binding module from Bifidobacterium bifidum
Source: FEBS Lett. 2025 Nov 7;600(4):504–14. doi: 10.1002/1873-3468.70217 (PMC12926856; doi:10.1002/1873-3468.70217)
Supplement: Supplementary file 1 — Fig. S1. Domain architecture of full‐length LnbB and a predicted structure by AlphaFold3. Fig. S2. Purification of LnbB‐CBM32. Fig. S3. TSA of LnbB‐CBM32 in the presence of various concentrations of LNB or GNB. Fig. S4. TSA of βSW‐LnbB‐CBM32 in the presence of various concentrations of LNB or GNB. Fig. S5. Anomalous difference Fourier maps. Fig. S6. Electron density map for LNB. Table S1. Crystallographic data statistics of LnbB‐CBM32. Table S2. Result of DALI structural similarity search. [file FEB2-600-504-s001.pdf]

Supporting Information

Structural insights into lacto-*N*-biose I recognition by a family 32 carbohydrate-binding module from *Bifidobacterium bifidum*

Xinzhe Zhang, Naoki Sunagawa, Toma Kashima, Kiyohiko Igarashi, Akimasa Miyanaga, and Shinya Fushinobu

Table S1. Crystallographic data statistics of LnbB-CBM32.
Table S2. Result of DALI structural similarity search.

Fig S1. Domain architecture of full-length LnbB and a predicted structure by AlphaFold3.
Fig S2. Purification of LnbB-CBM32.
Fig S3. TSA of LnbB-CBM32 in the presence of various concentrations of LNB or GNB.
Fig S4. TSA of βSW-LnbB-CBM32 in the presence of various concentrations of LNB or GNB.
Fig. S5. Anomalous difference Fourier maps.
Fig. S6. Electron density map for LNB.

Table S1. Crystallographic data statistics of LnbB-CBM32.

Dataset	Apo	LNB complex
**Data collection**[a]		
Beamline	SPring-8 BL45XU	KEK PF BL-1A
Wavelength (Å)	1.000	1.012
No. of crystals	6	1
No. of datasets	26	1
LCV (%)[b]	0.5	–
Space group	$P4_12_12$	$P22_12_1$
Unit cell (Å)	$a = b = 54.8$, $c = 202.9$	$a =56.5$, $b = 105.8$, $c = 122.1$
Resolution (Å)	50–2.00 (2.09–2.00)	48.54–2.05 (2.05–2.00)
Total reflections	1,002,597	340,305
Unique reflections	21,990	50,324
$CC_{1/2}$	0.978 (0.618)	0.991 (0.721)
Completeness (%)	99.9 (100.0)	100.0 (100.0)
Multiplicity	45.5 (40.4)	6.8 (7.0)
Mean $I/\sigma(I)$	8.84 (0.82)	7.8 (2.3)
R-factor	0.182 (1.41)	–
R-meas	0.184 (1.05)	–
R_{merge}	–	0.209 (0.927)
R_{pim}	–	0.086 (0.376)
Mol/ASU[c]	2	4
**Refinement**		
Resolution (Å)	48.25-2.00	48.54-2.00
No. of reflections	21,893	50,266
R/R_{free}	0.225/0.240	0.198/0.230
RMSD from ideal values		
Bond lengths (Å)	0.010	0.007
Bond angles (°)	1.77	0.788
Molprobity score	1.72	0.97
Clashscore	2.32	1.13
Ramachandran plot (%)		
Favored	91.64	97.19
Allowed	7.12	2.81
Outlier	0.00	0.00
**PDB code**	9WFH	9VAK

[a]Values in parentheses are for the highest resolution shell. Data collection statistics were calculated using XSCALE for the apo crystal and Aimless for the LNB complex crystal. [b]Linear cell variation. [c]Number of molecules per asymmetric unit.

Table S2. Result of DALI structural similarity search.

Protein	Source organism	PDB ID (chain)	Z score	RMSD (Å)	LALI[a]	%ID[b]
GH84 β-*N*-acetylhexosaminidase	*Clostridium perfringens*	2J1A (A)	20.3	1.9	139	29
AA5_2[c] galactose oxidase	*Fusarium* sp.	1K3I (A)	20.1	1.7	138	23
GH33 sialidase	*Micromonospora viridifaciens*	1WCQ (B)	19.9	2.0	142	25
AA5_2[c] galactose oxidase	*Hypomyces rosellus*	1GOF (A)	19.8	1.6	135	24
GH29 α-fucosidase	*Akkermansia muciniphila*	8AYR (A)	16.5	2.6	139	25

Analyzed using the DALI server (http://ekhidna2.biocenter.helsinki.fi/dali/). [a]Number of aligned residues. [b]Sequence identity. [c]Auxiliary Activities family 5, subfamily 2.

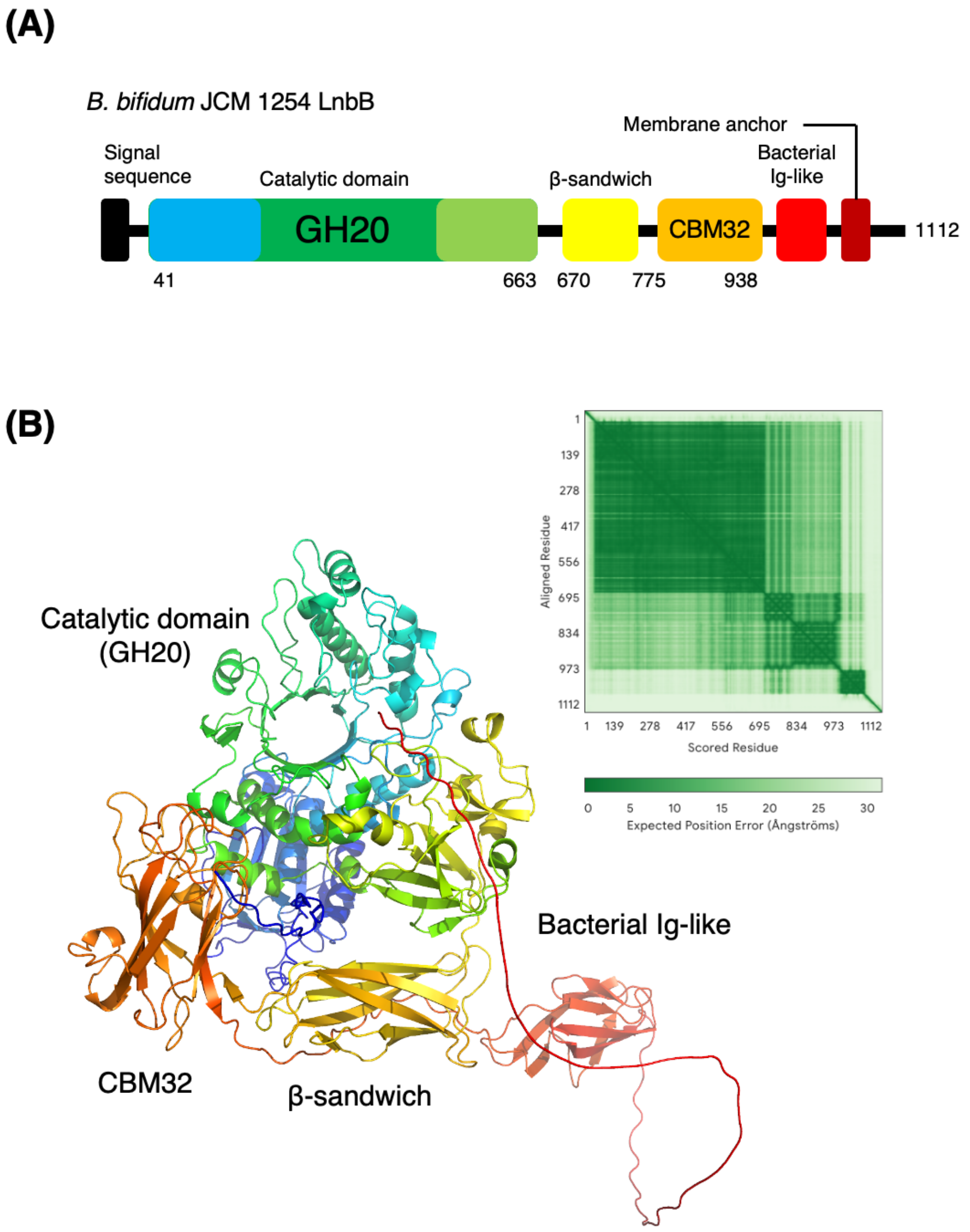

Fig S1. Detailed domain architecture of (A) full-length LnbB and (B) a predicted structure by AlphaFold3. The domain colors are matched between the two panels for comparison. The predicted aligned error plot is shown in (B).

(A)

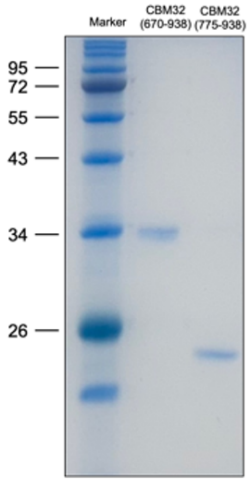

(B)

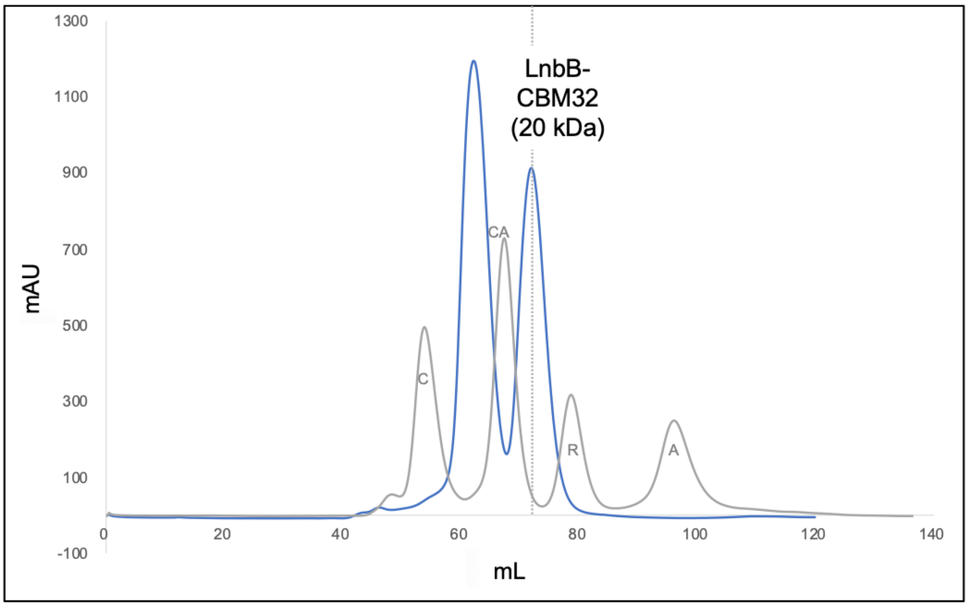

Fig S2. Purification of LnbB-CBM32. (A) SDS-PAGE of the protein size marker (left lane), LnbB-βSW-CBM32 (middle lane), and LnbB-CBM32 (right lane). (B) Elution profile for LnbB-CBM32 (blue line) from a size exclusion chromatography column. The left peak was a contaminated protein after the immobilized metal ion affinity chromatography purification. The column was calibrated separately using protein standards (gray line): C, conalbumin (75 kDa), CA, carbonic anhydrase (29 kDa), R, ribonuclease A (13.7 kDa), and A, aprotinin (6.5 kDa).

(A)

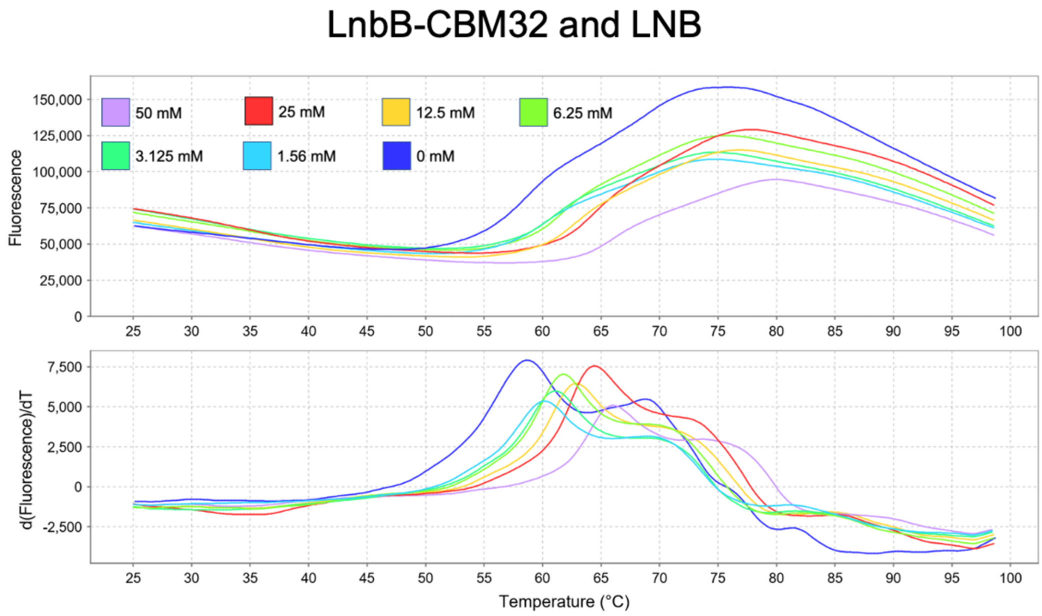

(B)

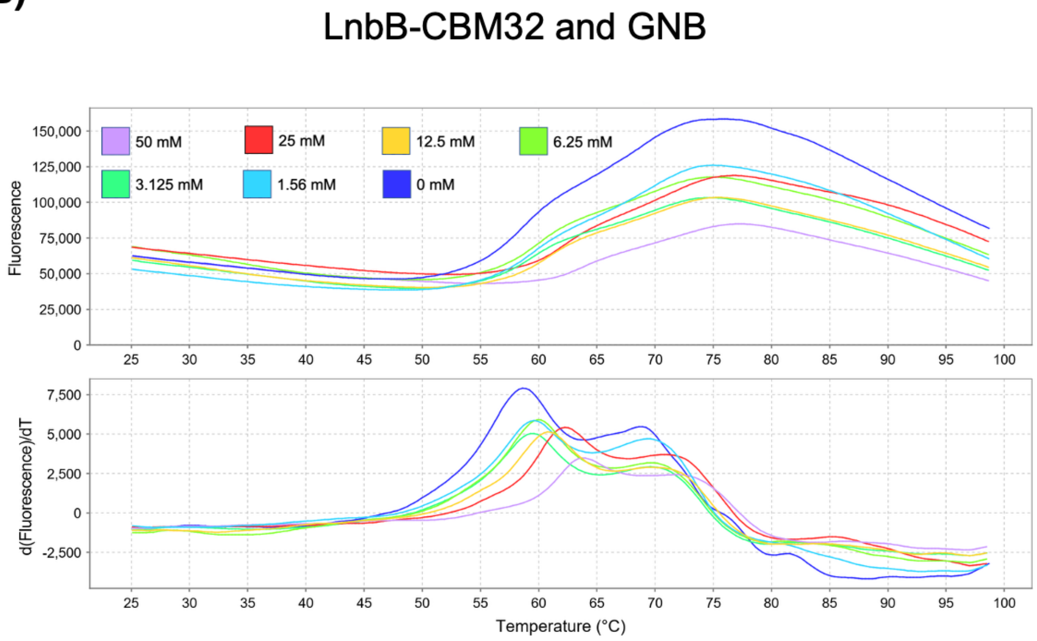

Fig S3. TSA of LnbB-CBM32 in the presence of various concentrations of (A) LNB or (B) GNB. Fluorescence and derivative melt curves are shown in the upper and lower panels, respectively. One of the triplicate sets of measurements is shown.

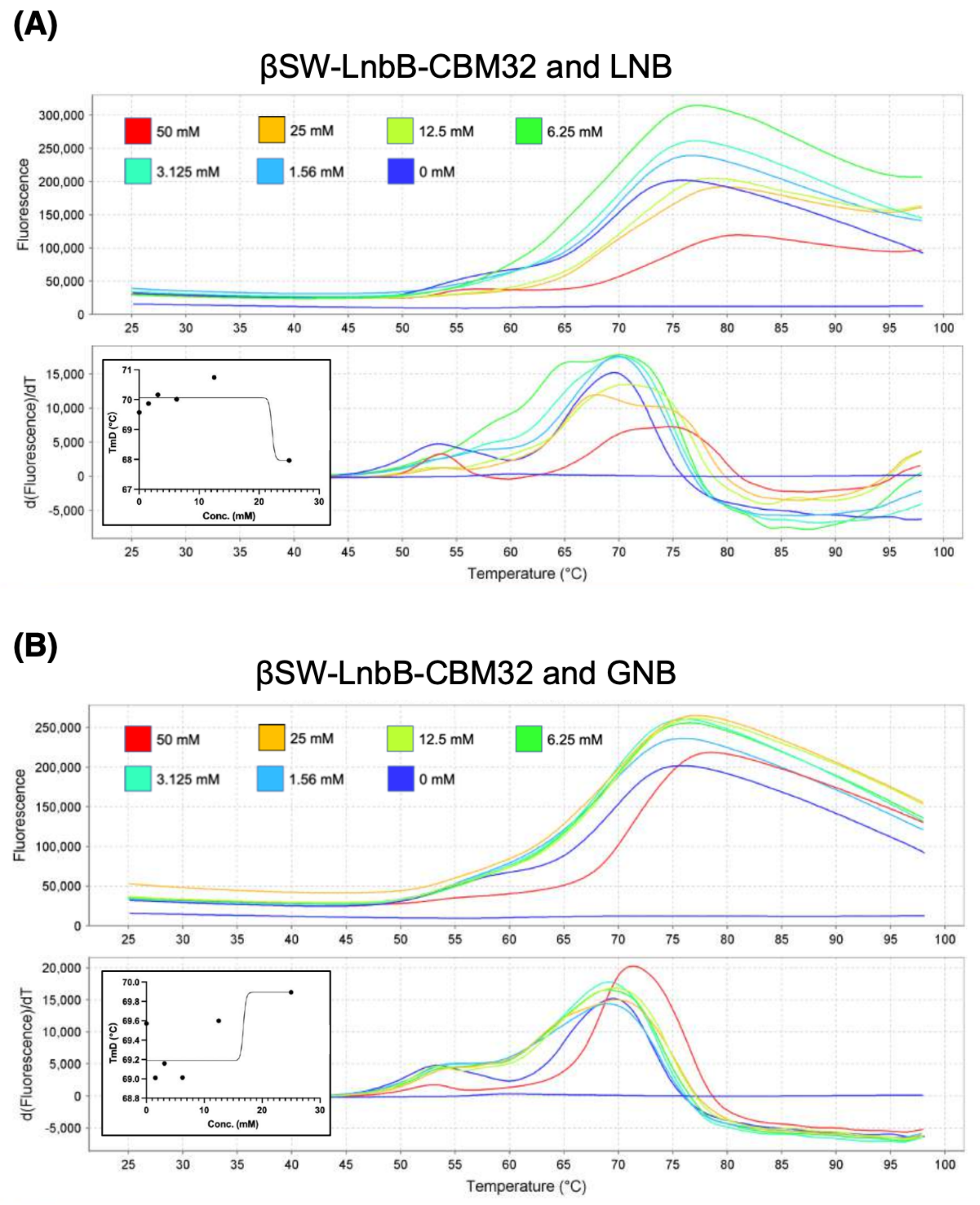

Fig S4. TSA of βSW-LnbB-CBM32 in the presence of various concentrations of (A) LNB or (B) GNB. Fluorescence and derivative melt curves are shown in the upper and lower panels, respectively.

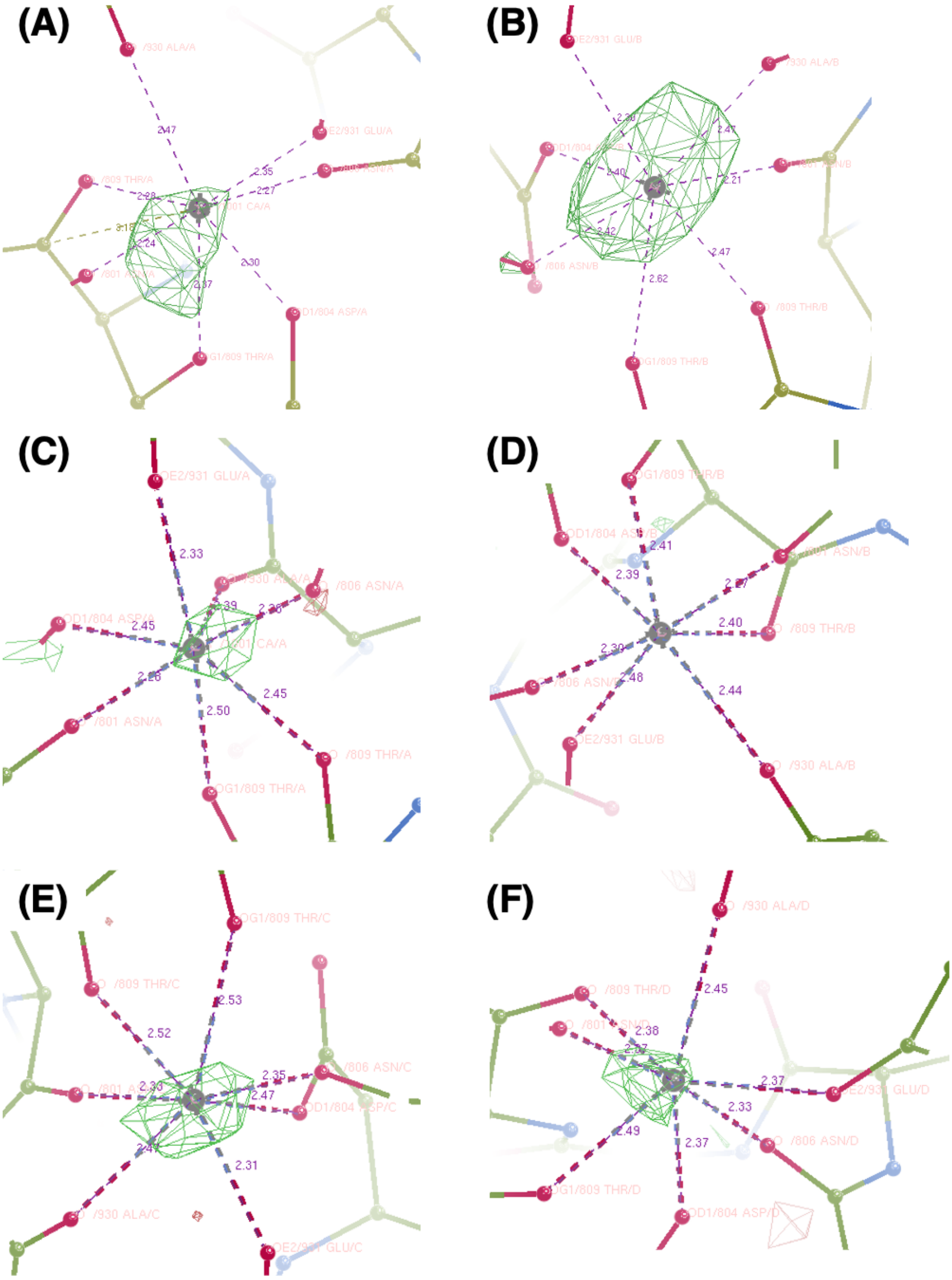

Fig. S5. Anomalous difference Fourier maps. Log-likelihood gradient maps (positive 3σ as green mesh and negative 3σ as red mesh) are shown for chains A (A) and B (B) in the apo structure, and for chains A (C), B (D), C (E), and D (F) in the LNB complex structure.

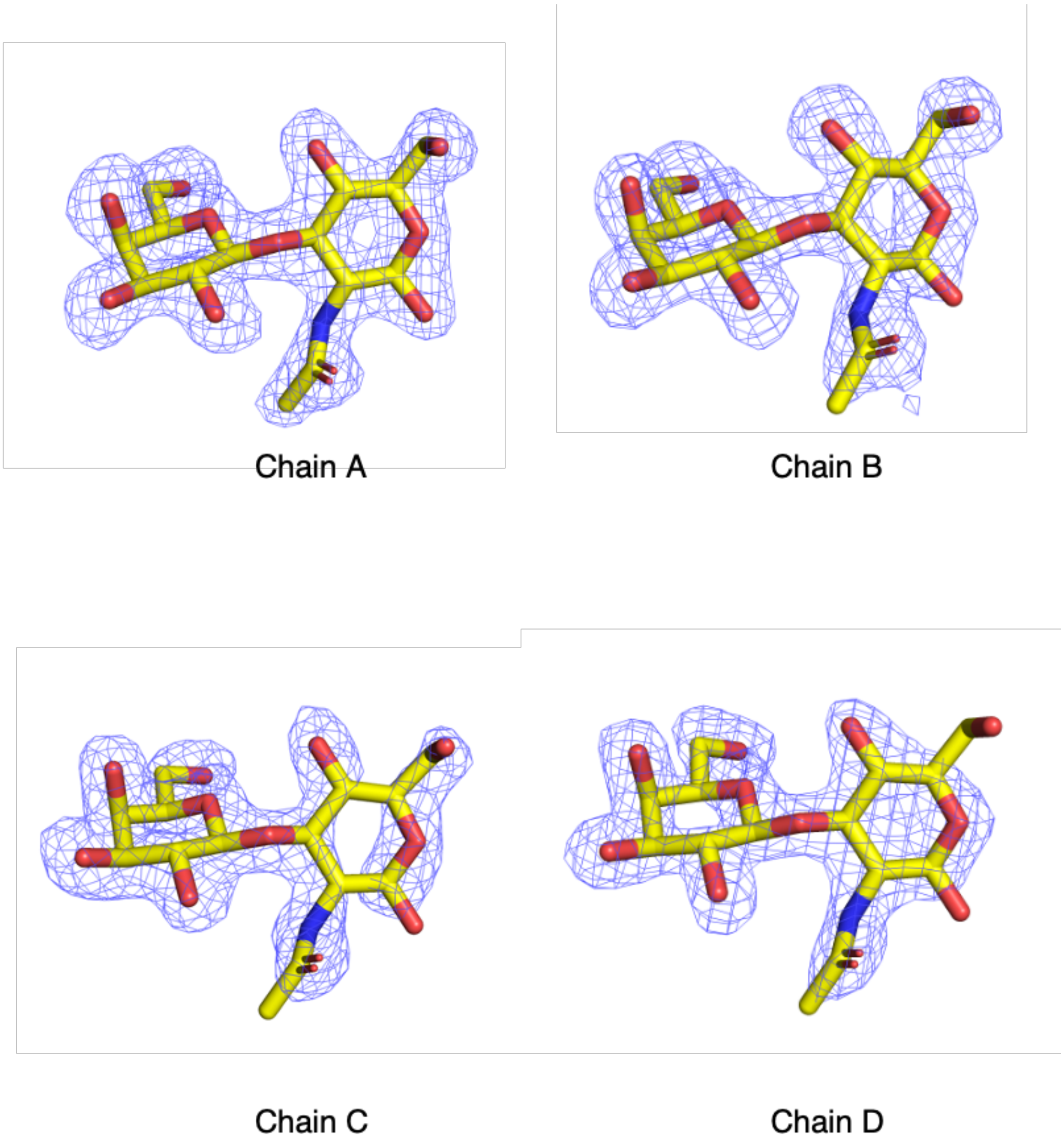

Fig. S6. Electron density maps for LNB. LNB molecules in each chain are shown with a Polder map (blue mesh, 3σ).
